# Efficient Convolution Network to Assist Breast Cancer Diagnosis and Target Therapy

**DOI:** 10.3390/cancers15153991

**Published:** 2023-08-06

**Authors:** Ching-Wei Wang, Kai-Lin Chu, Hikam Muzakky, Yi-Jia Lin, Tai-Kuang Chao

**Affiliations:** 1 Graduate Institute of Biomedical Engineering, National Taiwan University of Science and Technology, Taipei 106335, Taiwan; m11023103@mail.ntust.edu.tw (K.-L.C.); m11123801@mail.ntust.edu.tw (H.M.); 2 Department of Pathology, Tri-Service General Hospital, Taipei 11490, Taiwan; b93401052@ntu.edu.tw; 3 Institute of Pathology and Parasitology, National Defense Medical Center, Taipei 11490, Taiwan

**Keywords:** breast cancer metastases, histopathology, breast cancer target therapy, dilated soft label deep learning

## Abstract

**Simple Summary:**

Early detection and personalized treatment for breast cancer are vital for breast cancer patients survival. Computational pathology approaches can be employed by pathologists and cytologists to improve the efficiency and accuracy of breast cancer diagnosis and target therapy. With the recent development of machine learning and deep learning, there is an immense amount of optimism that this technology will eventually be able to handle difficulties that were previously unsolvable. Here, we developed an efficient deep learning method with a low computational cost to assist pathologists or cytologists with the task of detecting breast cancer metastases on H&E-stained WSIs and calculating HER2 and CEN17 signals for breast cancer anti-HER2 targeted therapy practically while minimizing individual judgment errors.

**Abstract:**

Breast cancer is the leading cause of cancer-related deaths among women worldwide, and early detection and treatment has been shown to significantly reduce fatality rates from severe illness. Moreover, determination of the human epidermal growth factor receptor-2 (HER2) gene amplification by Fluorescence in situ hybridization (FISH) and Dual in situ hybridization (DISH) is critical for the selection of appropriate breast cancer patients for HER2-targeted therapy. However, visual examination of microscopy is time-consuming, subjective and poorly reproducible due to high inter-observer variability among pathologists and cytopathologists. The lack of consistency in identifying carcinoma-like nuclei has led to divergences in the calculation of sensitivity and specificity. This manuscript introduces a highly efficient deep learning method with low computing cost. The experimental results demonstrate that the proposed framework achieves high precision and recall on three essential clinical applications, including breast cancer diagnosis and human epidermal receptor factor 2 (HER2) amplification detection on FISH and DISH slides for HER2 target therapy. Furthermore, the proposed method outperforms the majority of the benchmark methods in terms of IoU by a significant margin (p<0.001) on three essential clinical applications. Importantly, run time analysis shows that the proposed method obtains excellent segmentation results with notably reduced time for Artificial intelligence (AI) training (16.93%), AI inference (17.25%) and memory usage (18.52%), making the proposed framework feasible for practical clinical usage.

## 1. Introduction

In clinical pathology, pathologists manually determine the occurrence, form and severity of cancer and study the nuclear phenotype, tissue architecture and cytology among other parameters by examining tissue slides in order to assess the cancer staging and grading [1]. In clinical practice, examining tissue slides under a microscope is difficult, tiresome and prone to inter- and intra-observer variability [1,2]. Due to the recent developments in the scanning technologies that convert glass tissue slides to digital slides, digital pathology (DP) has fundamentally revolutionized the everyday work of pathologists [2]. DP can help in disease diagnosis by allowing for easy viewing and navigation of tissue slide images [1]. Furthermore, DP fosters cutting-edge research possibilities in image processing and computation to help automate cancer detection [3]. Recent advances in the field of computer vision and deep learning have it possible to detect sub-visual image information which may not be easily detected by the naked human eye. When applied for pathological images, deep learning methods extract useful characteristics from pathological images, resulting in better diagnosis and patient outcomes. Although, our previous efforts using deep learning have yielded promising results in applications to segmentation of cervical cancer [4], breast cancer [5], ovarian cancer [6,7] and HER2 status evaluation in breast cancer [8], some challenges limit its utility in practice. Firstly and most importantly, large computational cost of deep learning is the primary barrier in deploying these models in routine clinical practice. Secondly, when dealing with tissue or cell regions of interest, containing blurry or unclear boundaries, the performance of deep learning model deteriorates. To deal with the abovementioned issues, we propose an improved fully convolution network with low computing cost as an extended work of our previous studies [4,5,6,7,8,9], with three main improvements. Firstly, a dilated convolution is integrated into the proposed network to enlarge receptive fields for extracting multi-scale contextual information without losing resolution and greatly speeding up the model efficiently, hence reducing processing time and memory usage. Secondly, the FCN-32s architecture is replaced with the FCN-2s architecture to improve image segmentation results on data with blurry and unclear boundaries. Thirdly, we devise a soft weight softmax loss function to improve image segmentation performance of the model (see Section 3.2 for further details). In this study, we demonstrated the robustness and effectiveness of the proposed framework on three essential clinical applications, including breast cancer diagnosis and human epidermal receptor factor 2 (HER2) amplification detection on FISH and DISH slides for HER2 target therapy. The three clinical applications are explained as follows.

Breast cancer is the most frequent and lethal tumors in women across the world [10]. Even though the prognosis of patients with breast cancer is normally good but it worsens dramatically when the disease metastasizes [11]. Therefore, it is crucial to determine the presence of metastases in order to provide proper therapy and increase the chance of survival. Tumor, node and metastasis (TNM) staging criteria is formally used to determine the amount and spread of breast in the body of a patient. In routine clinical practice, the pathologists manually examine the glass slide containing a H&E-stained tissue section of the lymph node. Metastases are classified into three types depending upon the number of individual tumor cells or the diameter of clustered tumor cells: macro-metastases, micro-metastases or isolated tumor cells (ITC). The huge amount of tissue that must be inspected to find metastases is challenging for manual visual inspection in assessing lymph node status, and pathologists may overlook minor metastases.

The copy number of the HER2 gene is increased in approximately 20–30% of breast cancer patients, and determining the level of the HER2 receptor is important in current breast cancer diagnosis and treatment [12]. HER2-amplified tumors have an inferior prognosis in the absence of anti-HER2 treatment, but when administered HER2-targeting medicines such as trastuzumab, pertuzumab, and TDM-1, they are shown to significantly improve survival [13,14,15,16]. Every patient who has an IHC equivocal positive result (2+) must undergo FISH analysis to assess the HER2/CEN17 ratio and average HER2 copy number per nucleus in a minimum of 20 nuclei for anti-HER2 target treatments [17]. HER2 analysis by DISH has emerged as a viable alternative to FISH and has been FDA-approved [18]. Over the past 5+ years, DISH has replaced fluorescent methods in some laboratories [19]. Directly evaluating HER2 amplification status is tedious, laborious, and error-prone. Computerized clinical image diagnosis techniques are possibly the most effective sector of healthcare applications, as they can dramatically improve pathologists time efficiency and counting accuracy [20,21,22].

In this research, we built an efficient deep learning algorithm with low computing cost intended to assist cytologists or pathologists with the task of detecting breast cancer metastases on H&E-stained WSIs and calculating HER2 and CEN17 signals for breast cancer anti-HER2 targeted therapy practically. After that, we discuss related works in Section 2. In Section 3, the materials and method are reported in details. In Section 4, we conduct a comparison study of the proposed method with thirteen recently published deep learning models, including FCN [23], Modified FCN [4,5,6,7,9], SL-FCN [8], U-Net [24] + InceptionV4 [25], Ensemble of U-net with Inception-v4 [25], Inception-Resnet-v2 encoder [25], and ResNet-34 encoder [26], U-Net [24], SegNet [27], BCNet [28], CPN [29], SOLOv2 [30] and DeepLabv3+ [31] with three different backbones, including MobileNet [32], ResNet [26] and Xception [33]. In Section 5, we further conducted run time analysis. Lastly, discussions and conclusions are described in Section 6 and Section 7.

## 2. Related Works

### 2.1. Dilated Convolution

In recent years, a fully convolutional network (FCN) and its modified versions have been widely utilized for medical image segmentation tasks [4,5,6,7,9]. However, Minaee et al. [34] demonstrate that the FCN model is computationally expensive for real-time inference. Yu et al. [35] first introduced the concept of a dilated convolution for combining multiple levels contextual data effectively without sacrificing resolution and showed that for dense prediction simplifying the adapted network with dilated convolution can increase accuracy. DeepLabv3+ [31], a popular deep learning model, is also devised with dilated convolution in Atrous Spatial Pyramid Pooling (ASPP). To boost the model efficiency and accuracy, we integrated a dilated (a.k.a “atrous”) convolution into the proposed method, which accommodates an extra parameter called the dilation rate that affects the receptive fields of a convolution filter.

### 2.2. Segmentation Approaches

In recent years, due to the success of deep learning models in medical image analysis, there has been a significant amount of effort directed toward creating medical image segmentation algorithms utilizing deep learning models [9,23,30,31]. U-Net is introduced by Ronneberger et al. [24] and is commonly used for medical image segmentation. The U-Net architecture design comprises a contracting pathway to capture information and an expanding symmetrical path for accurate localization. Furthermore, a fully convolutional network (FCN) developed by Shelhamer et al. [23] is also used for medical image segmentation. To further improve the segmentation performance of FCN, researchers have developed a modified FCN-32s method and demonstrated the superior performance of modified FCN-32s in tumor segmentation of cervical cancer [4], breast cancer [5] and ovarian cancer [6,7]. Nishimura et al. [36] developed a weakly supervised cell instance segmentation approach that can separate individual cell areas under diverse scenarios using just approximate cell centroid locations as training data to decrease annotation costs. Rad et al. [37] proposed a fully convolutional deep learning models based on U-Net for trophectoderm segmentation in human embryo images. Raza et al. [38] proposed Micro-Net which is a fully convolutional deep learning framework for segmentation of cells, nuclei and glands in microscopic images. In this study, we present an improved and extended DSL-FCN2s deep learning model that achieves almost similar results as the previous effort [8] but takes less time and memory usage for training and inference for practical clinical usage. Here, we develop a proposed method and compare it with thirteen baseline deep learning methods, including FCN [23], Modified FCN [4,5,6,7,9], SL-FCN [8], U-Net [24] + InceptionV4 [25], Ensemble of U-net with Inception-v4 [25], Inception-Resnet-v2 encoder [25], and ResNet-34 encoder [26], U-Net [24], SegNet [27], YOLOv5 [39], BCNet [28], CPN [29], SOLOv2 [30] and DeepLabv3+ [31] with three different backbones, including MobileNet [32], ResNet [26] and Xception [33].

## 3. Materials and Methods

### 3.1. Materials

In this study, we collected three datasets, including one gigapixel WSI dataset and two microscopy datasets, from three institutions, including Tri-Service General Hospital, National Defense Medical Center and National Taiwan University Hospital, Taipei, Taiwan. The data distribution for training and testing is consistent with the associated studies [5,8,9] to ensure a fair comparison with the benchmark methods. The detailed information of three experimental datasets (see Table 1) is presented in the following sections.

#### 3.1.1. Breast Cancer Metastases WSI Dataset

The breast cancer metastases dataset [5] was acquired from The National Taiwan University Hospital and has been approved on 8 March 2019 by the research ethics committee B of the National Taiwan University Hospital (NTUH-REC 201810082RINB), containing 188 H&E and IHC CK(AE1/AE3)-stained lymph slides. The breast cancer dataset consists of 94 H&E-stained slides and 94 IHC CK(AE1/AE3)-stained WSIs. Breast cancer slide specimens containing lymphatic metastases were imaged utilizing a 3DHISTECH Pannoramic (3DHISTECH Kft., Budapest, Hungary) scanner with 20× objective magnification. The average size of the breast cancer WSI is 113,501 × 228,816 pixels. Qualified pathologists performed all of the annotations with the use of IHC biomarkers. The entire dataset was divided into three different subsets for training (60 slides or 63.8% from the entire dataset), validation (8 slides or 8.5% from the entire dataset) and testing (26 slides or 27.7% from the entire dataset).

#### 3.1.2. FISH Fluorescent Microscopy Dataset of Invasive Breast Cancer

The tissue bank of the Department of Pathology, Tri-Service General Hospital, National Defense Medical Center, Taipei, Taiwan, has provided the FISH fluorescent microscopy dataset [8] with ethical approvals acquired from the research ethics committee of the Tri-Service General Hospital (TSGHIRB No.1-107-05-171 and No.B202005070). The data was de-identified and utilized in retrospective research without affecting patient treatment. Digitized and de-identified slides of Dual-color FISH in breast infiltrating ductal carcinoma patients with HER2 IHC scores 2+ equivocal positive were obtained from January 2014 to December 2021 (a total of 200 FISH microscopy images). The FISH specimens were captured utilizing an Olympus microscope (Olympus, Japan) with 600× overall magnification. The average size of the FISH images is 1360 × 1024 pixels. The entire FISH dataset was split into three separate subsets for training, validation and testing, including 124 slides for training (60%), 14 slides for validation (7%) and 66 slides for testing (33%).

#### 3.1.3. DISH Light Microscopy Dataset of Invasive Breast Cancer

The DISH light microscopy dataset [8] was acquired from the tissue bank of the Department of Pathology, Tri-Service General Hospital, National Defense Medical Center, Taipei, Taiwan, and have been approved by the research ethics committee of the Tri-Service General Hospital (TSGHIRB No.1-107-05-171 and No.B202005070). De-identified, digitized images of dual-color DISH in ERBB2 IHC scores 2+ equivocal cases were obtained from January 2014 to December 2021. The DISH specimen slides were acquired by employing an Olympus microscope (Olympus, Japan) with 600× overall magnification. The average dimension of the DISH slides is 1360 × 1024 pixels. The entire set of DISH images was divided into three subsets for training (containing 37 slides, or 61.7% of the whole dataset), validation (containing 5 slides, or 8.3% of the whole dataset), and testing (containing 18 slides, or 30% of the whole dataset).

### 3.2. Proposed Method: Dilated Soft Label FCN2s

**Figure 1:**
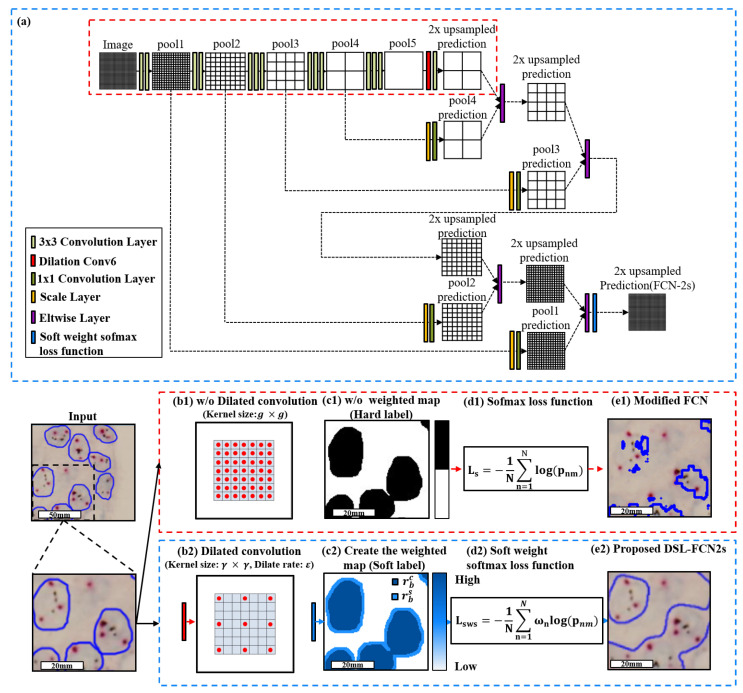
The overview of the proposed DSL-FCN2s architecture. (**a**) Modified FCN (red line) and Dilate Soft FCN2s (blue line) network architecture. (**b1**) original convolution of Conv6 convolution (**b2**) dilation Conv6 convolution. (**c1**) Hard label as the input for softmax loss function (**c2**) build the soft label and then obtain the soft-weight softmax loss input (the pixel weight $\omega_{m}$). (**d1**) The original loss function equation in the Modified FCN method, (**d2**) Our soft-weight softmax loss function equation used in our proposed method. (**e1**) Segmentation output result of the Modified FCN method, (**e2**) Segmentation output result of the proposed dilated soft label FCN2s method.

In this study, we propose an improved dilated soft-label fully convolution network with low computing cost as an extended work of our previous studies(Modified FCN) in applications to segmentation of cervical cancer [4], breast cancer [5], ovarian cancer [6,7] and HER2 status evaluation in breast cancer [8], with three main improvements. Firstly, to effectively combine multi-scale contextual information without compromising resolution, a dilated convolution is implemented into the proposed network. This is achieved by replacing the sixth convolution layer with a dilated convolution layer to greatly speed up the model efficiently and hence reduce processing time and memory usage. Secondly, the FCN-32s architecture is replaced with the FCN-2s architecture to enhance image segmentation outcomes on data with hazy or blurred cell borders. Thirdly, we devise a soft weight softmax loss function to improve the segmentation performance of the model. Figure 1 presents the overview of the proposed dilated soft-label FCN2s (DSL-FCN2s) architecture. For histopathology images, we propose dilated FCN2s (D-FCN2s) that generate precise segmentation results with much less time and memory usage for training and inference time.

#### 3.2.1. Proposed Dilate Soft-Label FCN Architecture

**Figure 2:**
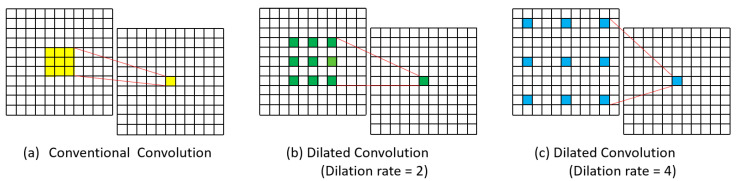
Visualization of conventional and dilated convolution. (**a**) Conventional convolution. (**b**) Dilated convolution kernels with dilation rate 2. (**c**) Dilated convolution kernels with dilation rate 4.

Firstly, to deal with the issue of large training time and GPU memory usage in training, the Modified FCN convolutional architecture is replaced with a dilation rate in the sixth convolutional layer, which has expensive parameters. Different from conventional convolution, dilated convolution has a distance (dilation rate ε) between each kernel element, allowing it to cover a larger area for extracting multi-scale contextual information. Figure 2 shows a comparison between (a) conventional convolution, (b) dilated convolution kernels with a dilation rate of 2 and (c) dilated convolution kernels with a dilation rate of 4.

Given the dilation rate ε = 3, the objective is to compute the kernel size of the dilation convolution (denoted as γ) when the output kernel size $\gamma^{\prime }$ has the same receptive field as the kernel size of the original sixth convolutional layer *g* in Modified FCN. The specific formula for obtaining the kernel size of the dilation convolution is formulated as:

(1) $$\gamma =\frac{g+\varepsilon -1}{\varepsilon }$$

(2) $$\gamma^{\prime }=\left(\gamma +\left(\gamma -1\right)\left(\varepsilon -1\right)\right)$$

However, magnifying the receptive field with no increase in computational cost can reduce about one-eighth of the parameters for training. For example, a γ×γ kernel with a dilation rate of ε will have the same size as $\gamma^{\prime }\times \gamma^{\prime }$ kernel while using only $\gamma^{2}$ parameters.

The output dimensions of the dilated convolution layer is expressed as follows:

(3) $$\left(\zeta_{fh},\zeta_{fw},\zeta_{fr}\right)=\left(\left\lceil \frac{\delta_{fh}+2P_{dconv6}-\gamma^{\prime }}{\xi }+1\right\rceil ,\left\lceil \frac{\delta_{fw}+2P_{dconv6}-\gamma^{\prime }}{\xi }+1\right\rceil ,\delta_{fc}\right)$$

where $\delta_{fh}$ and $\delta_{fw}$ are height and width of the input feature, respectively; $P_{dconv6}$ is the padding size of the Dilated Conv6 layer; ξ is the stride size; $\delta_{fc}$ is the number of input channels; $\zeta_{fh}$, $\zeta_{fw}$ and $\zeta_{fr}$ are height, width and number of output channels in the dilation convolution layer.

Secondly, to improve the medical image tumor segmentation results, FCN-32s is replaced with FCN-2s to obtain a large number of features that would be lost by using FCN-32s. Although, FCN-2s have a more complex architecture than FCN-32s which will result in more training and inference time. To deal with this issue, we have utilized the dilation rate in the sixth convolutional layer. Figure 1a and Table 2 present the comprehensive architecture of the proposed DSL-FCN2s.

Thirdly, we devise a soft-weight softmax loss function to enhance the segmentation performance of the model. We introduce additional weights, which are typically learned during training and help the model focus on more challenging regions or classes where accurate segmentation is crucial.

In this study, after applying dilation and erosion operations to the label, two values, namely $R^{o}={\left\{r_{b}^{o}\right\}}_{b=1,2,\ldots ,B}$ and $R^{c}={\left\{r_{b}^{c}\right\}}_{b=1,2,\ldots ,B}$ will be produced, the erosion area $R^{e}={\left\{r_{b}^{e}\right\}}_{b=1,2,\ldots ,B}$ and the dilation area $R^{d}={\left\{r_{b}^{d}\right\}}_{b=1,2,\ldots ,B}$ are described as follows.

(4) $$r_{b}^{e}=r_{b}^{a}\cap \left(∼r_{b}^{c}\right)$$

(5) $$r_{b}^{d}=r_{b}^{o}\cap \left(∼r_{b}^{a}\right)$$

where *b* denotes the number of annotations per image and $r_{b}^{a}$ is the original region in the *b*-th annotation.

However, the soft label regions $R^{s}={\left\{r_{b}^{s}\right\}}_{b=1,2,\ldots ,B}$ defined as the union of erosion areas $r_{b}^{e}$ and dilation areas $r_{b}^{d}$ which can be expressed as the following Equation:

(6) $$r_{b}^{s}=r_{b}^{e}\cup r_{b}^{d}$$

After generating the core regions of annotations $R^{c}$, cell boundary regions as $R^{s}$ and background regions, the soft weight $\omega_{\left(n\right)}$ for each pixel at position *n* is modeled using the following formulation:

(7) $$\omega_{\left(n\right)}=\left\{\begin{array}{cc} \Pi & ,n\in R^{c}\\ \Omega & ,n\in R^{s}\\ \phi & ,otherwise \end{array}\right.$$

where Π, Ω and ϕ is empirically determined (we use Π=2, Ω=1.5, and ϕ=1 in this study).

As shown in Equation (7), the loss weight formula is critical to guide the model’s attention during the training process. It assigns higher importance to the core regions of annotations, lowers the emphasis on boundary regions, and minimizes the effect of the background on the learning process. This way, the model is encouraged to concentrate on the most informative regions, leading to better segmentation performance.

In the training process, we employed the soft weight softmax loss function $L_{sws}$ in our proposed DSLFCN2s (see Figure 1d2). The soft weight softmax loss function is a variation of the cross-entropy loss function, where additional soft weights $\omega_{n}$ are introduced to modify the standard cross-entropy formulation. The soft weight softmax loss function can be formulated as follows:

(8) $$L_{sws}=-\frac{1}{N}\sum_{n=1}^{N}\omega_{n}\log\left(p_{nm}\right)$$

where *N* represents the total number of pixels of training data, $p_{nm}$ is the predicted probability of pixel *n* belonging to the target class *m* and $\omega_{n}$ denotes the soft weight value assigned to the pixel *n*.

(9) $$p_{nm}=\frac{e^{z_{nm}}}{\sum_{t=1}^{M}e^{z_{nt}}}$$

where *M*, $z_{nm}$ and $z_{mt}$ represent the total number of classes, the predicted score *z* for pixel *n* belongs to the target class *m* and the predicted score for pixel *n* belongs to the *t*-th class (where t∈[1,M]).

#### 3.2.2. Model Selection

Model selection approaches for deep learning algorithms have a strong connection to the mathematical optimization of a proper model selection parameter typically, such as k-fold cross-validation or leave-one-out cross-validation. It is the process of selecting an optimal model from a set of candidate models, derived by the training data. For model selection, we take a proportion ϵ of the training set $U_{train}={\left\{u_{train}^{\varpi }\right\}}_{\varpi =0}^{\varphi }$ as the validation set $U_{val}$ (we use $\epsilon =\frac{1}{9}$ in this study), which could be formulated as follows.

(10) $$U_{ts}={\left\{u_{pos}^{\alpha }\right\}}_{\alpha =0}^{\vartheta }\cup {\left\{u_{neg}^{\beta }\right\}}_{\beta =0}^{\eta }$$

(11) $$U_{val}={\left\{u_{pos}^{\alpha }\right\}}_{\alpha =\vartheta }^{\varrho }\cup {\left\{u_{neg}^{\beta }\right\}}_{\beta =\eta }^{\varphi -\varrho }$$

(12) $$U_{train}=U_{ts}\cup U_{val}=U_{pos}\cup U_{neg}$$

where $U_{ts}$ is a subset for training from $U_{train}$. $U_{pos}$, $U_{neg}$ and ϱ denote the positive samples of $U_{train}$, negative samples of $U_{train}$ and the number of the positive samples in the whole training set $U_{train}$, respectively. ϑ=⌊(1−ϵ)ϱ⌋ represents the number of the positive samples in $U_{ts}$ and η=⌊(1−ϵ)(φ−ϱ)⌋ is the number of the negative samples in $U_{ts}$.

Given a specified maximum number of training iterations and the number of *y* models to generate. For model selection, we tune the hyper-parameters, including learning rate, batch size, optimizer and training iteration to maximize the Dice Coefficient $Q_{\chi_{l}}={\left\{q_{\chi_{l}}\right\}}_{l=1}^{y}$ with model $\chi_{l}$ on the validation set $U_{val}$ and then select the best model $\chi_{l}^{*}$ with the highest Dice Coefficient $q_{\chi_{l}}^{*}$.

(13) $$q_{\chi_{l}}^{*}=\underset{l}{argmax}\left(q_{\chi_{l}}\right)$$

In summary, the hyper-parameters associated with the best model represent the optimal hyper-parameter configuration for that specific model architecture. This approach selects the best model and its corresponding hyper-parameter settings for the given task.

#### 3.2.3. WSI Processing Framework

To effectively deal with the huge dimension of WSIs, individual WSI W(a,b) was restructured as a patch-wise image data $D={\left\{D_{w,\psi }^{l}\left(i,j\right)\right\}}_{l=1}^{N}\in W\left(a,b\right)$, where *w* is the patch column index, ψ denotes patch row index, *i* represents patch horizontal subindex, *j* is patch vertical subindex and *l* denotes the image level. When l = N, α, β, *i*, and *j* were formulated as shown in Equation (14):

(14) $$\begin{array}{cc} w=\lfloor a/\alpha \rfloor , & \psi =\lfloor b/\beta \rfloor ,\\ i=a-w\times \alpha , & j=b-\psi \times \beta \end{array}$$

where α and β are the patch width and the patch height, respectively. The values w,ψ,i and *j* are in the range {0,⋯,ζ−1}, {0,⋯,η−1}, {0,⋯,α−1} and {0,⋯,β−1}, respectively; We utilized (α,β) = (512,512) in this study.

Initially, individual WSIs $w_{w,\psi }^{r}\left(i,j\right)$ were processed by Otsu’s method at the image level closest to the size of a unit tile (α,β). After that, each filtered tile is mapped back to the highest magnification level to effectively remove the background patches (tiles that have ≤70% tissue foreground), dramatically reducing the computational cost per WSI. The value of tissue foreground fraction r was calculated as follows:

(15) $$r=argmin_{l}\left(\zeta \times \eta \geq 1\wedge card\left(w^{l}\right)\geq \alpha \times \beta \right)$$

Subsequently, each filtered tile $w_{w,\psi }^{N}\left(i,j\right)$ was processed by the proposed DSL-FCN *H* to produce the tumor cells probability as demonstrated in Equation (16). The detailed architecture of the proposed DSL-FCN is presented in Section 3.2.1.

(16) $$p_{w,\psi }^{N}{\left(i,j\right)}^{c}=H\left(w_{w,\psi }^{N}\left(i,j\right)\right)$$

where c={0,⋯,C} represents the number of classes corresponding to the background, foreground, and target class, encoded in the entries c∈{0,1,2}, respectively.

A two-dimensional pixel-wise class map was generated as the index of the cell type that had the maximum probability of the pixel described as follows.

(17) $$s_{w,\psi }^{N}\left(i,j\right)=argmax_{c}\left(\left(p_{w,\psi }^{N}{\left(i,j\right)}^{c}\right)\right)$$

Finally, the output segmentation results of target class $T=\{t_{w,\psi }^{N}\left(i,j\right)\}$ were generated using Equation (18) based on class map $s_{w,\psi }^{N}\left(i,j\right)$. Equation (18) suppressed the foreground information, generating the target information as the output.

(18) $$t_{w,\psi }^{N}\left(i,j\right)=\left\{\begin{array}{cc} I_{w,\psi }\left(i,j\right) & ,\;s_{w,\psi }^{N}\left(i,j\right)>1\\ \phi & ,otherwise \end{array}\right.$$

where ϕ is a null set.

#### 3.2.4. Implementation Details

In the training process, we used VGG16 model as a baseline model and utilized the stochastic gradient descent (SGD) optimizer. During training, the patches are selected in a randomized manner from the training set using a batch size of one. Next, the proposed model is optimized with a base learning rate, weight decay and momentum of $1\times 10^{-10}$, $5\times 10^{-4}$ and 0.99, respectively. Furthermore, the benchmark methods were developed and trained based on the standard parameters provided in the literature.

## 4. Results

This section compares the proposed method with thirteen state-of-the-art benchmark methods on the task of detecting breast cancer metastases on H&E-stained WSIs and calculating HER2 and CEN17 signals on FISH and DISH slides for HER2 target therapy. In addition, this section also provided statistical evaluation to compare the proposed method with the baseline approaches based on Fisher’s Least Significant Difference (LSD) tests utilizing SPSS software [40].

### 4.1. Quantitative Evaluation with Statistical Analysis in Breast Cancer Metastases Dataset

**Figure 3:**
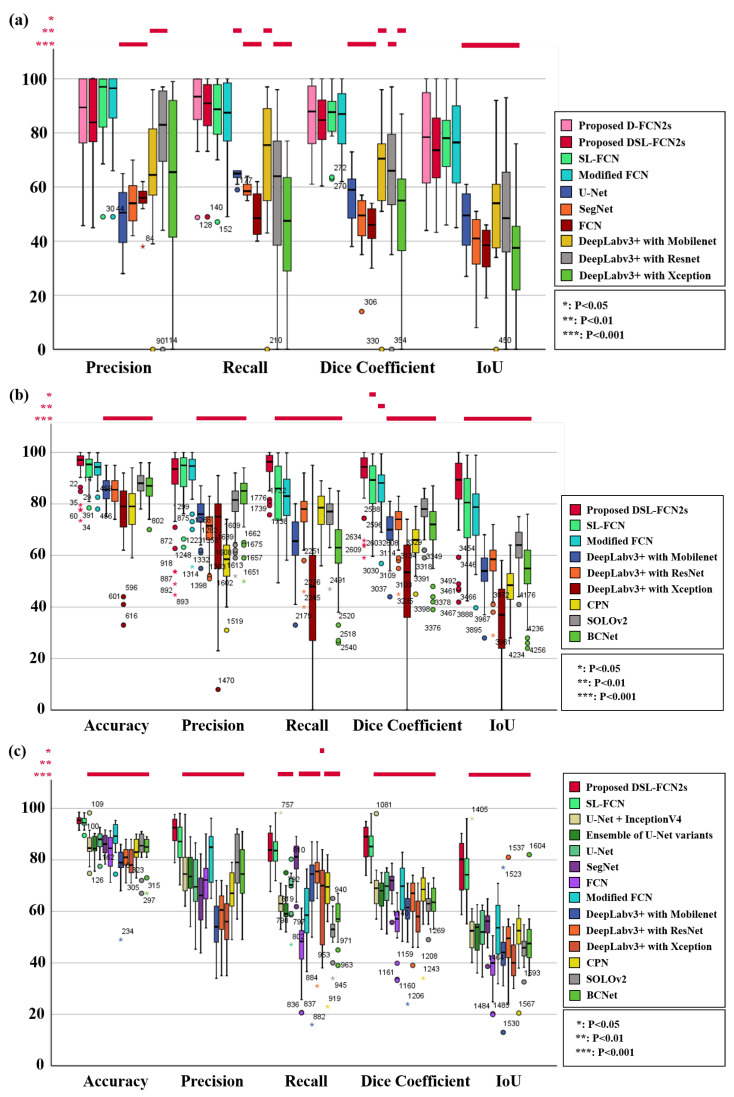
Boxplots of segmentation performance three breast cancer datasets, including (**a**) breast cancer metastases dataset, (**b**) FISH breast dataset and (**c**) DISH breast dataset, using the proposed method and the state-of-the-art deep learning methods.

**Figure 4:**
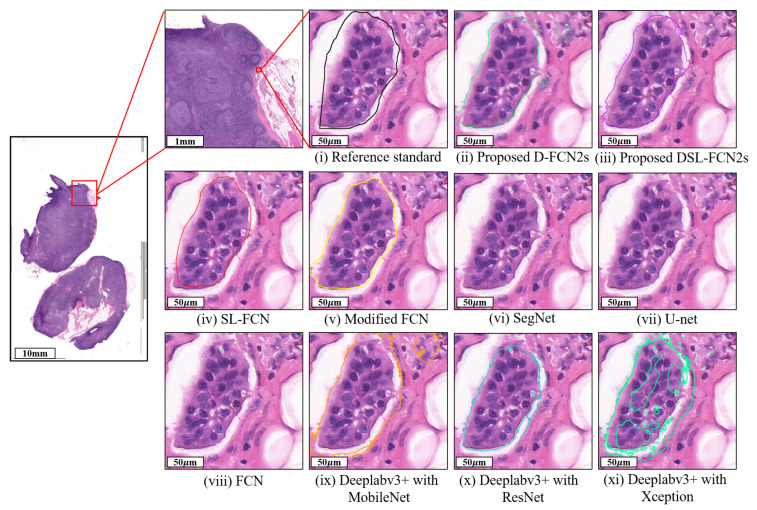
Qualitative segmentation results of the proposed DSL-FCN2s and the state-of-the-art deep learning models for segmentation of breast cancer metastases in H&E-stained WSIs.

The quantitative evaluation results show that the proposed D-FCN2s achieves a precision of 87.56 ± 16.67%, recall of 88.95 ± 15.85%, dice coefficient of 86.40 ± 13.36% and IoU of 78.13 ± 19.56% while the proposed DSL-FCN2s obtains a precision of 82.37 ± 17.78%, recall of 87.20 ± 13.90%, dice coefficient of 82.80 ± 12.23% and IoU of 72.35 ± 17.84% for segmentation of breast metastases on H&E stained WSIs (see Table 3a). Even for a larger number of patch samples from gigantic WSI, our proposed D-FCN2s and DSL-FCN2s methods still obtain highly remarkable performance in terms of precision, recall, dice coefficient and IoU. In comparison with the state-of-the-art deep learning methods, the proposed D-FCN2s performs significantly better than the majority of baseline approaches (i.e., six out of nine) in terms of IoU (*p* < 0.001); in terms of recall and dice coefficient, the proposed method outperformed the six out of nine benchmark methods with statistical significance (*p* < 0.01); in terms of the precision, the proposed method significantly outperformed the five out of nine benchmark methods (*p* < 0.01) (see Figure 3a and Supplementary Table S1). In addition, Figure 4 presents the qualitative segmentation results for the segmentation of breast cancer metastases comparing the proposed D-FCN2s with the baseline approaches. We can see that the proposed approach produces segmentation results consistent with the reference standard generated by expert pathologists.

### 4.2. Quantitative Evaluation with Statistical Analysis in FISH Breast Dataset

**Figure 5:**
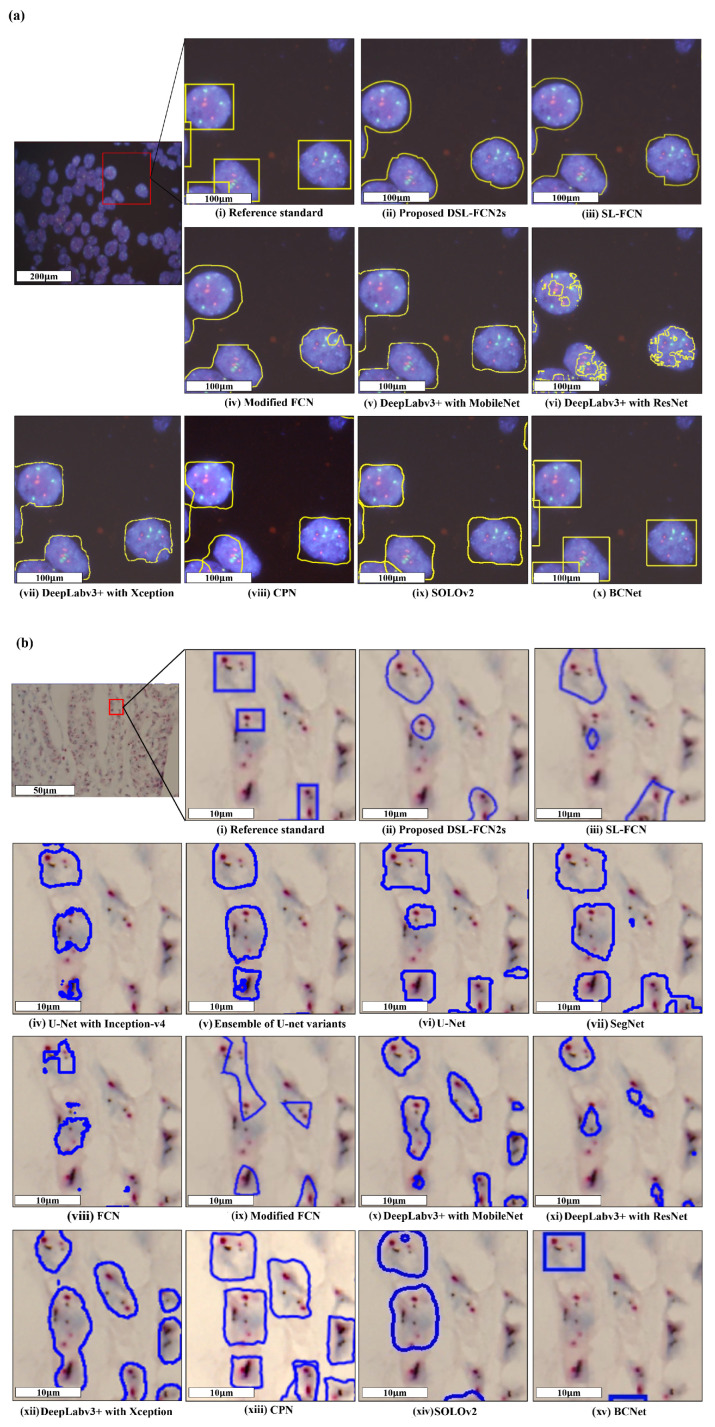
Qualitative segmentation results of the proposed DSL-FCN2s method and the benchmark approaches for segmentation of HER2 amplification in (**a**) DISH breast dataset and (**b**) FISH breast dataset. The red boxes indicate the zoomed-in part of the FISH and DISH original images; the yellow and blue boxes represent the prediction results of the proposed method in DISH and FISH datasets, respectively.

The quantitative evaluation results show that the proposed DSL-FCN2s achieves an accuracy of 95.46 ± 5.61%, precision of 89.30 ± 12.80%, recall of 94.76 ± 5.54%, dice coefficient of 91.55 ± 9.26% and IoU of 85.56 ± 13.83% for segmentation of HER2 amplification in FISH dataset (see Table 3b). In comparison with the state-of-the-art deep learning methods, the proposed DSL-FCN2s performs significantly better than all the benchmark methods in terms of recall and IoU with statistical significance (*p* < 0.001); in terms of dice coefficient, the proposed method is significantly better than the seven out of eight benchmark methods (*p* < 0.01); in terms of the accuracy, the proposed method outperformed six out of eight baseline methods with statistical significance (*p* < 0.001); in terms of the precision, the proposed method outperformed the six out of eight benchmark methods with statistical significance (*p* ≤ 0.001) (see Figure 3b and Supplementary Table S2). Figure 5a presents the qualitative segmentation results for segmentation of HER2 overexpression comparing the proposed DSL-FCN2s with the baseline approaches. It can be shown that the proposed method generates segmentation results that are appropriate with the reference standard determined by competent pathologists.

### 4.3. Quantitative Evaluation with Statistical Analysis in DISH Breast Dataset

The quantitative evaluation results show that the proposed DSL-FCN2s achieves an accuracy of 95.33 ± 1.89%, precision of 90.81 ± 6.04%, recall of 83.84 ± 7.26%, dice coefficient of 87.08 ± 6.08% and IoU of 77.60 ± 9.31% for segmentation of HER2 amplification in DISH dataset (see Table 3c). For DISH dataset, in terms of the accuracy, precision, dice coefficient and IoU, the proposed method is shown to be significantly better than 12 out of 13 state-of-the-art deep learning models (*p* < 0.001); for the recall, the proposed method outperformed the 11 out of 13 benchmark baselines with statistical significance (*p* < 0.05). (see Figure 3c and Supplementary Table S3). Figure 5b presents the qualitative segmentation results for segmentation of HER2 overexpression comparing the proposed DSL-FCN2s with the baseline approaches. Based on the segmentation results, the proposed method is shown to be identical with the reference standard produced by experienced pathologists.

## 5. Run Time Analysis and Ablation Study

In this section, we conduct two experiments to further compare the effectiveness and the efficiency of the baseline modified FCN model [4,5,6,7,9], the proposed DSL-FCN model using FISH breast cancer dataset with image resolution 1360 × 1024. For the first experiment, in the evaluation of the effectiveness, the results show that the proposed DSL-FCN2s consistently achieve the best performance in accuracy, precision, recall, and Dice Coefficient as shown in Table 4. To further investigate the contributions made by the proposed method in computational efficiency, we examine the run time analysis, including training time, memory usage and inference time, the number of parameters used in a single layer and the total number of parameters used for each model.

For the FISH and DISH datasets, the proposed method and the baseline methods are trained and tested on an NVIDIA GeForce GTX 1080 Ti GPU with 32 GB memory, respectively. As shown in Table 5, the results show that the computing cost in the training time, memory usage (1 MiB = 1.048576 MB), inference time, conv6 parameter, and total parameters of the proposed method are greatly reduced by 16.93%, 18.52%, 17.25%, 81.60%, and 62.48%, respectively.

Overall, our proposed method has demonstrated higher effectiveness and better efficiency with the improvement of considerably reducing training and inference time, memory usage and the number of parameters used in the FISH and DISH datasets applications.

## 6. Discussion

The application of computerized image processing in pathology could rapidly and precisely determine and quantify particular cell types, as well as quantitatively assess histological characteristics and morphological abnormalities [22]. Quantitative image assessment methods also allow for the data collecting from slide specimens that would otherwise be inaccessible during the routine microscopic inspection [41]. In this study, we developed an efficient deep learning algorithm with low computing cost intended to assist cytologists or pathologists in three essential clinical applications, including breast cancer diagnosis and detection of HER2 amplification on FISH and DISH slides for HER2 target therapy. Adequate diagnosis of breast cancer metastases and HER2 status is necessary for determining the appropriate treatment strategy. Anti-HER2 therapy has been demonstrated to be an effective strategy for the treatment of HER2-positive breast cancer [42]. HER2 overexpression has also been associated with ovary, endometrium, fallopian tube, gastric and prostate cancers [43,44,45]. Anti-HER2 therapies are now part of the care standard for HER2-amplified gastric cancer [46,47]. HER2 may also be a potential therapeutic target for quiescent prostate cancer [48]. Despite the fact that HER2 status in cancers of the female reproductive system has been explored for more than 20 years, the determination of HER2 gene status has not been widely recognized as a prognostic biomarker for response to anti-HER2 treatment in gynecologic cancers, unlike in the breast and the digestive system [49].

Artificial intelligence (AI) has recently shown significant benefits in medical image analysis considering of the rapid growth of deep learning methods, decreased testing turnaround along with the development of accurate and highly reproducible tissue-derived readouts lowering inter-pathologist variation [50,51,52,53]. In recent years, the advent of deep learning has emerged as a promising solution for the automatic analysis of medical images to improve diagnosis and precision oncology [20]. Thus, precise HER2 status determination is crucial for guiding therapy solutions. The HER2/CEN17 ratio and the average number of HER2 copies per nucleus (at least 20 nuclei) serve as the primary determinants of positive and negative amplification status. However, visual counting alone is easily prone to errors and difficult to reproduce in existing algorithms. Automated medical image diagnostic methods are arguably the most successful field in medical applications, which can greatly improve the time efficiency for the pathologist’s analysis and the accuracy of counting in a large number of clinical samples [20,21,22]. Therefore, an automated diagnostic method based on AI has the potential to overcome the limitations of manual assessment procedures [54,55,56,57]. Deep learning takes less than one second to analyze FISH or DISH images, the time for automatic report generation is significantly shorter than manual visual assessment. The main problem of this study is the difficulty in predicting the HER2 gene amplification status in part of FISH or DISH images of relatively low quality characterized by weak signals or overlapping nuclei with masking some signals. To overcome these limitations, we would need to improve the image resolution quality and increase the number of pathologists that provide annotations. The application of deep learning may provide a new method of FISH or DISH image and warrant further validation in a larger population-based study for practical use in clinical specimens in future work. In this study, we develop a highly efficient fully convolution network with low computing cost to aid in breast cancer target therapy and breast cancer diagnosis.

## 7. Conclusions

The experimental results demonstrate that the proposed DSL-FCN2s achieves a precision of 87.56%, recall of 88.95% and Dice Coefficient of 86.40% for segmentation of breast cancer metastases on H&E-stained WSIs. For FISH and DISH datasets, the proposed DSL-FCN2s achieves an accuracy of 95.46%, precision of 89.30%, recall of 94.76% and Dice Coefficient of 91.55% and accuracy of 95.33%, precision of 90.81%, recall of 83.84% and Dice Coefficient of 87.08% for segmentation of HER2 amplification on FISH and DISH breast datasets, respectively. We recommend using D-FCN2s for histopathology images and DSL-FCN2s for cytology, FISH and DISH images as DSL-FCN2s generates precise segmentation results on datasets containing cells with unclear boundaries. The proposed fully convolution network proves to be more objective, accurate, and independent than the present manual interpretation results for the detection of breast cancer metastases and anti-HER2 target therapy. Furthermore, in statistical analysis, the proposed method outperforms the majority of the benchmark methods in terms of IoU by a significant margin (p<0.001) on three different clinical applications. Importantly, run time analysis shows that the proposed method obtains excellent segmentation results with notably reduced time for AI training (16.93%), AI inference (17.25%) and memory usage (18.52%), making the proposed framework feasible for practical clinical usage. The ablation study and run time analysis demonstrate that the proposed method not only produces precise segmentation results but also takes less time and memory usage for training and inference time. In addition, the proposed deep learning-based approach that eradicates human error-related misclassifications alongside cuts down AI inference time, improving accuracy and reproducibility, which can be further validated in larger population-based research to help clinicians in the future. 

## Figures and Tables

**Table 1:** Detailed information of experimental datasets.

Dataset	Hospital	Cancer Type	Scanner/ Imaging System	Overall Magnification	Size (pixels)	Slides			
						Total	Training	Validation	Testing
H&E-stained WSI dataset [5]	National Taiwan University Hospital	Breast cancer	3DHISTECH Pannoramic	200×	113,501 × 228,816	94	60(63.8%)	8(8.5%)	26(27.7%)
FISH fluorescent microscopy dataset [8]	Tri-Service General Hospital National Defense Medical Center	Breast cancer	Olympus	600×	1360 × 1024	200	120(60%)	14(7%)	66(33%)
DISH light microscopy dataset [8]	Tri-Service General Hospital National Defense Medical Center	Breast cancer	Olympus	600×	1360 × 1024	60	37(61.7%)	5(8.3%)	18(30%)

**Table 2:** The comprehensive architecture of the proposed DSL-FCN2s.

Layer	Features (Train)	Features (Inference)	Kernel Size	Stride	Dilation
Input	512 × 512 × 3	512 × 512 × 3	-	-	-
Conv1_1	710 × 710 × 64	710 × 710 × 64	3 × 3	1	-
relu1_1	710 × 710 × 64	710 × 710 × 64	-	-	-
Conv1_2	710 × 710 × 64	710 × 710 × 64	3 × 3	1	-
relu1_2	710 × 710 × 64	710 × 710 × 64	-	-	-
Pool1	355 × 355 × 64	355 × 355 × 64	2 × 2	2	-
Scale	355 × 355 × 3	355 × 355 × 3	-	-	-
Convolution	355 × 355 × 64	355 × 355 × 64	1 × 1	-	-
Conv2_1	355 × 355 × 128	355 × 355 × 128	3 × 3	1	-
relu2_1	355 × 355 × 128	355 × 355 × 128	-	-	-
Conv2_2	355 × 355 × 128	355 × 355 × 128	3 × 3	1	-
relu2_2	355 × 355 × 128	355 × 355 × 128	-	-	-
Pool2	178 × 178 × 128	178 × 178 × 128	2 × 2	2	-
Scale	178 × 178 × 128	178 × 178 × 128	-	-	-
Convolution	178 × 178 × 3	178 × 178 × 3	1 × 1	-	-
Conv3_1	178 × 178 × 256	178 × 178 × 256	3 × 3	1	-
relu3_1	178 × 178 × 256	178 × 178 × 256	-	-	-
Conv3_2	178 × 178 × 256	178 × 178 × 256	3 × 3	1	-
relu3_2	178 × 178 × 256	178 × 178 × 256	-	-	-
Conv3_3	178 × 178 × 256	178 × 178 × 256	3 × 3	1	-
relu3_3	178 × 178 × 256	178 × 178 × 256	-	-	-
Pool3	89 × 89 × 256	89 × 89 × 256	2 × 2	2	-
Scale	89 × 89 × 256	89 × 89 × 256	-	-	-
Convolution	89 × 89 × 3	89 × 89 × 3	1 × 1	-	-
Conv4_1	89 × 89 × 512	89 × 89 × 512	3 × 3	1	-
relu4_1	89 × 89 × 512	89 × 89 × 512	-	-	-
Conv4_2	89 × 89 × 512	89 × 89 × 512	3 × 3	1	-
relu4_2	89 × 89 × 512	89 × 89 × 512	-	-	-
Conv4_3	89 × 89 × 512	89 × 89 × 512	3 × 3	1	-
relu4_3	89 × 89 × 512	89 × 89 × 512	-	-	-
Pool4	45 × 45 × 512	45 × 45 × 512	2 × 2	2	-
Scale	45 × 45 × 512	45 × 45 × 512	-	-	-
Convolution	45 × 45 × 3	45 × 45 × 3	1 × 1	-	-
Conv5_1	45 × 45 × 512	45 × 45 × 512	3 × 3	1	-
relu5_1	45 × 45 × 512	45 × 45 × 512	-	-	-
Conv5_2	45 × 45 × 512	45 × 45 × 512	3 × 3	1	-
relu5_2	45 × 45 × 512	45 × 45 × 512	-	-	-
Conv5_3	45 × 45 × 512	45 × 45 × 512	3 × 3	1	-
relu5_3	45 × 45 × 512	45 × 45 × 512	-	-	-
Pool5	23 × 23 × 512	23 × 23 × 512	2 × 2	2	-
Dilation Conv6	17 × 17 × 4096	17 × 17 × 4096	$\gamma^{\prime }$ × $\gamma^{\prime }$	1	ε
relu6 + Drop6	17 × 17 × 4096	17 × 17 × 4096	-	-	-
Conv7	17 × 17 × 4096	17 × 17 × 4096	1 × 1	1	-
relu7 + Drop7	17 × 17 × 4096	17 × 17 × 4096	-	-	-
Conv8	17 × 17 × N	17 × 17 × N	1 × 1	1	-
Deconv1	36 × 36 × N	36 × 36 × N	4 × 4	2	-
Crop1	36 × 36 × N	36 × 36 × N	-	-	-
Eltwise	36 × 36 × N	36 × 36 × N	-	-	-
Deconv2	74 × 74 × N	74 × 74 × N	4 × 4	2	-
Crop2	74 × 74 × N	74 × 74 × N	-	-	-
Eltwise	74 × 74 × N	74 × 74 × N	-	-	-
Deconv3	150 × 150 × N	150 × 150 × N	4 × 4	2	-
Crop3	150 × 150 × N	150 × 150 × N	-	-	-
Eltwise	150 × 150 × N	150 × 150 × N	-	-	-
Deconv4	302 × 302 × N	320 × 320 × N	4 × 4	2	-
Crop4	302 × 302 × N	302 × 302 × N	-	-	-
Eltwise	302 × 302 × N	302 × 302 × N	-	-	-
Deconv5	606 × 606 × N	606 × 606 × N	4 × 4	2	-
Crop5	512 × 512 × N	512 × 512 × N	-	-	-
Soft weight softmax loss	512 × 512 × N	512 × 512 × N	-	-	-
Output Class Map	512 × 512 × 1	512 × 512 × 1	-	-	-

Dilation kernel size γ = 3; the dilation rate ε = 3.

**Table 3:** Quantitative segmentation results of the proposed method and the benchmark methods on three breast cancer datasets, including (a) breast metastases dataset, (b) FISH breast dataset and (c) DISH breast dataset. The top-ranked quantitative segmentation results based on dice coefficient value are represented in bold format.

**(a) Breast Metastases WSI Dataset (Histopathology)**											
**Method**		**Precision**		**Recall**		**Dice Coefficient**		**IoU**		**Rank Dice** **Coefficient**	
**Proposed D-FCN2s**		**87.56 ± 16.67**		**88.95 ± 15.85**		**86.40 ± 13.36**		**78.13 ± 19.56**		**1**	
Proposed DSL-FCN2s		82.37 ± 17.78		87.20 ± 13.90		82.80 ± 12.23		72.35 ± 17.84		4	
SL-FCN [8]		88.83 ± 16.13		85.48 ± 15.39		85.23 ± 11.94		75.89 ± 17.25		2	
Modified FCN [4,5,6,7,9]		89.17 ± 16.21		83.67 ± 16.85		84.42 ± 12.78		74.92 ± 18.83		3	
DeepLabv3+ [31] with Mobilenet [32]		64.33 ± 26.22		68.25 ± 27.77		64.08 ± 24.11		50.42 ± 22.96		5	
DeepLabv3+ [31] with Resnet [26]		75.33 ± 28.64		58.42 ± 29.00		62.17 ± 25.95		48.75 ± 25.11		6	
DeepLabv3+ [31] with Xception [33]		61.33 ± 35.45		44.00 ± 26.12		48.00 ± 26.24		34.42 ± 21.39		8	
U-Net [24]		48.58 ± 11.65		64.25 ± 2.26		56.42 ± 9.50		47.33 ± 11.48		7	
SegNet [27]		54.75 ± 9.10		58.83 ± 2.82		46.25 ± 12.48		38.00 ± 12.91		9	
FCN [23]		55.17 ± 6.18		50.00 ± 8.15		45.08 ± 7.89		36.33 ± 8.67		10	
**(b) FISH Breast Dataset**											
**Method**	**Accuracy**		**Precision**		**Recall**		**Dice Coefficient**		**IoU**		**Rank Dice** **Coefficient**
**Proposed DSL-FCN2s**	**95.46 ± 5.61%**		**89.30 ± 12.80%**		**94.76 ± 5.54%**		**91.55 ± 9.26%**		**85.56 ± 13.83%**		**1**
SL-FCN [8]	93.54 ± 5.24%		91.75 ± 8.27%		83.52 ± 13.15%		86.98 ± 9.85%		78.22 ± 14.73%		2
Modified FCN [4,5,6,7,9]	93.38 ± 4.46%		91.90 ± 7.87%		82.13 ± 10.99%		86.41 ± 8.38%		76.97 ± 12.50%		3
DeepLabv3+ [31] with Mobilenet [32]	85.17 ± 5.18%		75.53 ± 6.14%		64.94 ± 9.99%		69.36 ± 7.27%		53.55 ± 8.08%		7
DeepLabv3+ [31] with Resnet [26]	85.06 ± 5.23%		69.79 ± 7.30%		76.44 ± 9.28%		72.52 ± 6.62%		57.29 ± 7.65%		5
DeepLabv3+ [31] with Xception [33]	76.83 ± 11.67%		66.35 ± 19.82%		45.27 ± 24.82%		47.55 ± 20.44%		33.73 ± 15.58%		9
CPN [29]	77.67 ± 8.38%		57.55 ± 8.46%		76.95 ± 8.03%		65.35 ± 6.72%		48.46 ± 7.37%		8
SOLOv2 [30]	88.11 ± 4.48%		79.55 ± 8.01%		75.86 ± 6.60%		77.308 ± 5.82%		62.94 ± 7.45%		4
BCNet [28]	85.98 ± 5.58%		83.27 ± 8.11%		62.36 ± 12.08%		70.55 ± 9.77%		54.80 ± 10.79%		6
**(c) DISH Breast Dataset**											
**Method**	**Accuracy**		**Precision**		**Recall**		**Dice Coefficient**		**IoU**		**Rank Dice** **Coefficient**
**Proposed DSL-FCN2s**	**95.33 ± 1.89%**		**90.81 ± 6.04%**		**83.84 ± 7.26%**		**87.08 ± 6.08%**		**77.60 ± 9.31%**		**1**
SL-FCN [8]	94.64 ± 2.23%		86.78 ± 8.16%		83.78 ± 6.42%		85.14 ± 6.61%		74.67 ± 10.05%		2
U-Net [24]+InceptionV4 [25]	85.41 ± 5.25%		74.65 ± 9.90%		64.46 ± 9.57%		68.94 ± 8.92%		53.35 ± 12.17%		5
Ensemble of U-Net variants ${}^{\iota }$	84.82 ± 4.38%		74.39 ± 9.56%		61.28 ± 5.82%		66.89 ± 5.85%		51.69 ± 6.96%		7
U-Net [24]	86.89 ± 4.25%		70.40 ± 10.90%		69.09 ± 7.45%		69.13 ± 6.93%		52.97 ± 7.78%		4
SegNet [27]	86.17 ± 3.92%		65.71 ± 10.84%		79.00 ± 8.46%		70.74 ± 5.68%		55.00 ± 6.59%		3
FCN [23]	83.75 ± 5.89%		72.55 ± 10.05%		45.71 ± 12.25%		54.23 ± 9.77%		37.75 ± 8.71%		14
Modified FCN [4,5,6,7,9]	89.05 ± 5.26%		82.12 ± 9.48%		59.42 ± 11.96%		68.30 ± 9.99%		52.68 ± 11.51%		6
DeepLabv3+ [31] with Mobilenet [32]	77.33 ± 8.51%		55.06 ± 9.59%		69.50 ± 16.74%		59.78 ± 10.57%		44.00 ± 12.18%		12
DeepLabv3+ [31] with Resnet [26]	80.89 ± 4.56%		59.00 ± 9.16%		73.28 ± 11.80%		64.17 ± 9.19%		48.56 ± 12.00%		9
DeepLabv3+ [31] with Xception [33]	78.72 ± 5.15%		56.00 ± 9.34%		63.61 ± 14.77%		57.89 ± 7.68%		40.67 ± 7.65%		13
CPN [29]	83.61 ± 5.23%		67.39 ± 8.02%		67.22 ± 13.21%		66.33 ± 10.09%		50.33 ± 10.06%		8
SOLOv2 [30]	84.78 ± 6.47%		79.11 ± 10.24%		52.44 ± 7.21%		62.22 ± 5.35%		45.34 ± 5.45%		11
BCNet [28]	83.72 ± 5.74%		73.61 ± 11.42%		57.06 ± 7.18%		63.50 ± 6.40%		48.50 ± 10.85%		10

^*ι*^ An ensemble hybrid model consisting of U-Net with Inception-v4 [25], U-Net with Inception-ResNet-v2 encoder [25] and, (c) U-Net with ResNet-34 encoder [26].

**Table 4:** Quantitative results for the ablation study. Quantitative results for the ablation study when using different network structure with FISH breast dataset. The top-ranked quantitative segmentation result based on dice coefficient rank is represented in bold format.

FISH Breast Dataset						
**Method**	**Accuracy**	**Precision**	**Recall**	**Dice Coefficient**	**IoU**	**Rank Dice** **Coefficient**
**Proposed DSL-FCN2s**	**95.46 ± 5.61%**	**89.30 ± 12.80%**	**94.76 ± 5.54%**	**91.55 ± 9.26%**	**85.56 ± 13.83%**	**1**
Propoesd DSL-FCN2s w/o model selection	93.67 ± 4.92%	91.89 ± 7.53%	83.32 ± 11.19%	87.13 ± 8.83%	78.20 ± 13.15%	2
SL-FCN [8]	93.54 ± 5.24%	91.75 ± 8.27%	83.52 ± 13.15%	86.98 ± 9.85%	78.22 ± 14.73%	3
Modified FCN + Dilated convolution + soft label weight loss	89.98 ± 8.04%	92.70 ± 6.71%	69.09 ± 20.63%	77.49 ± 17.09%	66.00 ± 20.26%	6
Modified FCN + Dilated convolution	92.93 ± 5.05%	91.59 ± 7.93%	80.57 ± 14.18%	85.14 ± 10.67%	75.46 ± 14.68%	5
Modified FCN [4,5,6,7,9]	93.38 ± 4.46%	91.90 ± 7.87%	82.13 ± 10.99%	86.41 ± 8.38%	76.97 ± 12.50%	4

**Table 5:** Run time analysis for computational efficiency. Runtime analysis for the ablation study when using different network structure with FISH breast dataset.

FISH Breast Dataset					
**Method**	**Training Time**	**Memory Usage**	**Inference Time**	**Conv6 Parameter**	**Total Parameter**
Proposed DSL−FCN2s	4 h 15 min(−16.93%)	2846 MiB(− 18.52%)	0.489 s(−17.25%)	18,878,464(−81.6%)	50.39 M(−62.48%)
Proposed DSL−FCN2s w/o model selection	4 h 9 min(−18.89%)	2846 MiB(−18.52%)	0.495 s(−16.24%)	18,878,464(−81.6%)	50.39 M(−62.48%)
SL−FCN [8]	5 h 10 min(+0.97%)	3493 MiB	0.563 s(−4.73%)	102,764,544	134.31 M
Modified FCN + Dilated convolution + soft label weight loss	4 h 9 min(−18.89%)	2535 MiB(−27.42%)	0.505 s(−14.55%)	18,878,464(−81.6%)	50.42 M(−62.45%)
Modified FCN + Dilated convolution	4 h 7 min(−19.54%)	2535 MiB(−27.42%)	0.515 s(−12.85%)	18,878,464(−81.6%)	50.42 M(−62.45%)
Modified FCN ${}^{\Psi }$ [4,5,6,7,9]	5 h 7 min	3493 MiB	0.591 s	102,764,544	134.31 M

^Ψ^ Modified FCN is the baseline for the runtime analysis.

## Data Availability

The data that support the findings of this study are available from the corresponding author upon reasonable request.

## Supplementary Materials

The following supporting information can be downloaded at: https://www.mdpi.com/article/10.3390/cancers15153991/s1, Table S1: Multiple comparisons for segmentation of breast cancer metastases on WSIs; Table S2: HER2 amplification on FISH breast dataset; Table S3: DISH breast dataset.
