# Supplementary material for: Efficient Convolution Network to Assist Breast Cancer Diagnosis and Target Therapy
Source: Cancers (Basel). 2023 Aug 6;15(15):3991. doi: 10.3390/cancers15153991 (PMC10416960; doi:10.3390/cancers15153991)
Supplement: Supplementary file 1 [file cancers-15-03991-s001.zip › cancers-2532630-supplementary.pdf]

Supplementary Materials: Efficient Convolution Network to Assist Breast Cancer Diagnosis and Target Therapy

Ching-Wei Wang [1,*], Kai-Lin Chu [1], Hikam Muzakky [1], Yi-Jia Lin [2,3] and Tai-Kuang Chao [2,3,*]

Table S1. Multiple comparisons for segmentation of breast cancer metastases on WSI dataset : LSD test.

LSD Multiple Comparsion							
						95%C.I.	
Measurement	(I) Method	(J) Method	Mean Difference(I-J)	Std. Error	Sig.	Lower Bound	Upper Bound
Precision	Proposed D-FCN2s	Proposed DSL-FCN2s	5.19	8.34	0.535	-11.33	21.71
		Proposed SL-FCN [1]	-1.27	8.49	0.881	-18.1	15.56
		Modified FCN [2–6]	-1.61	8.49	0.85	-18.44	15.23
		U-Net [7]	***38.98	8.49	<0.001	22.14	55.81
		SegNet [8]	***32.81	8.49	<0.001	15.97	49.64
		FCN [9]	***32.39	8.49	<0.001	15.56	49.23
		Deeplabv3+ [10] with MobileNet [11]	**23.23	8.49	0.007	6.39	40.06
		Deeplabv3+ [10] with Xception [12]	**26.23	8.49	0.003	9.39	43.06
		Deeplabv3+ [10] with ResNet [13]	12.23	8.49	0.153	-4.61	29.06
Recall	Proposed D-FCN2s	Proposed DSL-FCN2s	1.75	7.48	0.815	-13.07	16.57
		Proposed SL-FCN [1]	3.47	7.62	0.65	-11.63	18.58
		Modified FCN [2–6]	5.28	7.62	0.49	-9.82	20.39
		U-Net [7]	**24.7	7.62	0.002	9.59	39.8
		SegNet [8]	***30.11	7.62	<0.001	15.01	45.22
		FCN [9]	***38.95	7.62	<0.001	23.84	54.05
		Deeplabv3+ [10] with MobileNet [11]	**20.7	7.62	0.008	5.59	35.8
		Deeplabv3+ [10] with Xception [12]	***44.95	7.62	<0.001	29.84	60.05
		Deeplabv3+ [10] with ResNet [13]	***30.53	7.62	<0.001	15.43	45.64
Dice coefficient	Proposed D-FCN2s	Proposed DSL-FCN2s	3.6	6.96	0.606	-10.18	17.39
		Proposed SL-FCN [1]	1.17	7.09	0.869	-12.88	15.21
		Modified FCN [2–6]	1.98	7.09	0.78	-12.06	16.03
		U-Net [7]	***29.98	7.09	<0.001	15.94	44.03
		SegNet [8]	***40.15	7.09	<0.001	26.1	54.19
		FCN [9]	***41.31	7.09	<0.001	27.27	55.36
		Deeplabv3+ [10] with MobileNet [11]	**22.31	7.09	0.002	8.27	36.36
		Deeplabv3+ [10] with Xception [12]	***38.4	7.09	<0.001	24.35	52.44
		Deeplabv3+ [10] with ResNet [13]	**24.23	7.09	0.001	10.19	38.28
IoU	Proposed D-FCN2s	Proposed DSL-FCN2s	5.78	7.48	0.441	-9.05	20.61
		Proposed SL-FCN [1]	2.24	7.62	0.77	-12.87	17.35
		Modified FCN [2–6]	3.22	7.62	0.674	-11.89	18.33
		U-Net [7]	***30.8	7.62	<0.001	15.69	45.91
		SegNet [8]	***40.13	7.62	<0.001	25.02	55.24
		FCN [9]	***41.8	7.62	<0.001	26.69	56.91
		Deeplabv3+ [10] with MobileNet [11]	***27.72	7.62	<0.001	12.61	42.83
		Deeplabv3+ [10] with Xception [12]	***43.72	7.62	<0.001	28.61	58.83
		Deeplabv3+ [10] with ResNet [13]	***29.38	7.62	<0.001	14.27	44.49

The mean difference is significant at the level of *0.05, **0.01 and ***0.001.

Table S2. Multiple comparisons for segmentation of HER2 amplification on FISH breast dataset : LSD test.

LSD Multiple Comparsion							
						95%C.I.	
Measurement	(I) Method	(J) Method	Mean Difference(I-J)	Std. Error	Sig.	Lower Bound	Upper Bound
Accuracy	Proposed DSL-FCN2s	SL-FCN [1]	1.92	1.15	0.095	-0.34	4.17
		Modified FCN [2–6]	2.08	1.15	0.07	-0.17	4.33
		DeepLabv3+ [10]with Mobilenet [11]	***10.29	1.15	<0.001	8.04	12.54
		DeepLabv3+ [10]with ResNet [13]	***10.4	1.15	<0.001	8.14	12.65
		DeepLabv3+ [10]with Xception [12]	***18.62	1.15	<0.001	16.37	20.88
		CPN [14]	***17.79	1.15	<0.001	15.54	20.04
		SOLOv2 [15]	***7.35	1.15	<0.001	5.1	9.6
		BCNet [16]	***9.47	1.15	<0.001	7.22	11.73
Precision	Proposed DSL-FCN2s	SL-FCN [1]	-2.46	1.82	0.177	-6.02	1.11
		Modified FCN [2–6]	-2.61	1.82	0.152	-6.17	0.96
		DeepLabv3+ [10]with Mobilenet [11]	***13.77	1.82	<0.001	10.2	17.34
		DeepLabv3+ [10]with ResNet [13]	***19.51	1.82	<0.001	15.94	23.08
		DeepLabv3+ [10]with Xception [12]	***22.95	1.82	<0.001	19.38	26.52
		CPN [14]	***31.75	1.82	<0.001	28.19	35.32
		SOLOv2 [15]	***9.75	1.82	<0.001	6.19	13.32
		BCNet [16]	***6.03	1.82	0.001	2.46	9.59
Recall	Proposed DSL-FCN2s	SL-FCN [1]	***11.24	2.16	<0.001	7	15.47
		Modified FCN [2–6]	***12.63	2.16	<0.001	8.4	16.87
		DeepLabv3+ [10]with Mobilenet [11]	***29.82	2.16	<0.001	25.59	34.06
		DeepLabv3+ [10]with ResNet [13]	***18.32	2.16	<0.001	14.09	22.56
		DeepLabv3+ [10]with Xception [12]	***49.49	2.16	<0.001	45.26	53.72
		CPN [14]	***17.81	2.16	<0.001	13.58	22.04
		SOLOv2 [15]	***18.9	2.16	<0.001	14.67	23.13
		BCNet [16]	***32.4	2.16	<0.001	28.17	36.63
Dice Coefficient	Proposed DSL-FCN2s	SL-FCN [1]	*4.57	1.78	0.011	1.07	8.07
		Modified FCN [2–6]	**5.14	1.78	0.004	1.64	8.64
		DeepLabv3+ [10]with Mobilenet [11]	***22.18	1.78	<0.001	18.68	25.68
		DeepLabv3+ [10]with ResNet [13]	***19.03	1.78	<0.001	15.53	22.53
		DeepLabv3+ [10]with Xception [12]	***44	1.78	<0.001	40.5	47.5
		CPN [14]	***26.2	1.78	<0.001	22.7	29.69
		SOLOv2 [15]	***14.17	1.78	<0.001	10.67	17.66
		BCNet [16]	***21	1.78	<0.001	17.5	24.5
IoU	Proposed DSL-FCN2s	SL-FCN [1]	***7.61	1.98	<0.001	3.73	11.5
		Modified FCN [2–6]	***8.59	1.97	<0.001	4.72	12.47
		DeepLabv3+ [10]with Mobilenet [11]	***32.02	1.97	<0.001	28.15	35.89
		DeepLabv3+ [10]with ResNet [13]	***28.28	1.97	<0.001	24.4	32.15
		DeepLabv3+ [10]with Xception [12]	***51.84	1.97	<0.001	47.96	55.71
		CPN [14]	***37.1	1.97	<0.001	33.23	40.97
		SOLOv2 [15]	***22.62	1.97	<0.001	18.75	26.5
		BCNet [16]	***30.76	1.97	<0.001	26.89	34.63

The mean difference is significant at the level of *0.05, **0.01 and ***0.001.

Table S3. Multiple comparisons for segmentation of HER2 amplification on DISH breast dataset : LSD test.

LSD Multiple Comparsion							
						95%C.I.	
Measurement	(I) Method	(J) Method	Mean Difference(I-J)	Std. Error	Sig.	Lower Bound	Upper Bound
		SL-FCN [1]	0.69	1.71	0.686	-2.68	4.06
		U-Net [7]+InceptionV4 [17]	***11.23	1.71	<0.001	7.86	14.6
		Ensemble of U-Net variants$^{\iota}$	***10.51	1.71	<0.001	7.14	13.88
		U-Net [7]	***8.44	1.71	<0.001	5.07	11.81
		SegNet [8]	***9.16	1.71	<0.001	5.79	12.53
		FCN [9]	***11.58	1.71	<0.001	8.21	14.95
Accuracy	Proposed DSL-FCN2s	Modified FCN [2–6]	***6.28	1.71	<0.001	2.91	9.65
		DeepLabv3+ [10]with Mobilenet [11]	***18	1.71	<0.001	14.63	21.37
		DeepLabv3+ [10]with ResNet [13]	***14.44	1.71	<0.001	11.07	17.81
		DeepLabv3+ [10]with Xception [12]	***16.61	1.71	<0.001	13.24	19.98
		CPN [14]	***11.72	1.71	<0.001	8.35	15.09
		SOLOv2 [15]	***10.55	1.71	<0.001	7.18	13.92
		BCNet [16]	***11.61	1.71	<0.001	8.24	14.98
		SL-FCN [1]	4.03	3.15	0.202	-2.17	10.24
		U-Net [7]+InceptionV4 [17]	***17.25	3.15	<0.001	11.05	23.46
		Ensemble of U-Net variants$^{\iota}$	***16.42	3.15	<0.001	10.21	22.63
		U-Net [7]	***20.41	3.15	<0.001	14.2	26.62
		SegNet [8]	***25.1	3.15	<0.001	18.9	31.31
		FCN [9]	***18.25	3.15	<0.001	12.05	24.46
Precision	Proposed DSL-FCN2s	Modified FCN [2–6]	***8.69	3.15	<0.001	2.48	14.89
		DeepLabv3+ [10]with Mobilenet [11]	***35.75	3.15	<0.001	29.55	41.96
		DeepLabv3+ [10]with ResNet [13]	***31.81	3.15	<0.001	25.6	38.02
		DeepLabv3+ [10]with Xception [12]	***34.81	3.15	<0.001	28.6	41.02
		CPN [14]	***23.42	3.15	<0.001	17.21	29.63
		SOLOv2 [15]	***11.7	3.15	<0.001	5.49	17.9
		BCNet [16]	***17.2	3.15	<0.001	10.99	23.4
		SL-FCN [1]	0.06	3.43	0.985	-6.7	6.83
		U-Net [7]+InceptionV4 [17]	***21.29	3.43	<0.001	14.53	28.05
		Ensemble of U-Net variants$^{\iota}$	***22.57	3.43	<0.001	15.8	29.33
		U-Net [7]	***14.75	3.43	<0.001	7.99	21.51
		SegNet [8]	4.84	3.43	0.16	-1.92	11.6
		FCN [9]	***38.14	3.43	<0.001	31.38	44.9
Recall	Proposed DSL-FCN2s	Modified FCN [2–6]	***24.43	3.43	<0.001	17.67	31.19
		DeepLabv3+ [10]with Mobilenet [11]	***14.34	3.43	<0.001	7.58	21.11
		DeepLabv3+ [10]with ResNet [13]	***10.57	3.43	<0.001	3.8	17.33
		DeepLabv3+ [10]with Xception [12]	*20.23	3.43	0.02	13.47	27
		CPN [14]	***16.62	3.43	<0.001	9.86	23.38
		SOLOv2 [15]	***31.4	3.43	<0.001	24.64	38.16
		BCNet [16]	***26.79	3.43	<0.001	20.03	33.55
		SL-FCN [1]	1.94	2.59	0.454	-3.16	7.04
		U-Net [7]+InceptionV4 [17]	***19.7	2.59	<0.001	14.59	24.8
		Ensemble of U-Net variants$^{\iota}$	***20.2	2.59	<0.001	15.09	25.3
		U-Net [7]	***17.95	2.59	<0.001	12.85	23.05
		SegNet [8]	***16.35	2.59	<0.001	11.24	21.45
		FCN [9]	***32.86	2.59	<0.001	27.76	37.96
Dice Coefficient	Proposed DSL-FCN2s	Modified FCN [2–6]	***18.78	2.59	<0.001	13.68	23.89
		DeepLabv3+ [10]with Mobilenet [11]	***27.31	2.59	<0.001	22.21	32.41
		DeepLabv3+ [10]with ResNet [13]	***22.92	2.59	<0.001	17.82	28.02
		DeepLabv3+ [10]with Xception [12]	***29.2	2.59	<0.001	24.09	34.3
		CPN [14]	***20.75	2.59	<0.001	15.65	25.85
		SOLOv2 [15]	***24.86	2.59	<0.001	19.76	29.96
		BCNet [16]	***23.58	2.59	<0.001	18.48	28.68
		SL-FCN [1]	2.92	3.06	0.341	-3.11	8.96
		U-Net [7]+InceptionV4 [17]	***26.56	3.06	<0.001	20.53	32.6
		Ensemble of U-Net variants$^{\iota}$	***25.9	3.06	<0.001	19.87	31.94
		U-Net [7]	***24.62	3.06	<0.001	18.59	30.66
		SegNet [8]	***22.6	3.06	<0.001	16.56	28.64
		FCN [9]	***39.85	3.06	<0.001	33.81	45.88
IoU	Proposed DSL-FCN2s	Modified FCN [2–6]	***24.91	3.06	<0.001	18.88	30.95
		DeepLabv3+ [10]with Mobilenet [11]	***33.6	3.06	<0.001	27.56	39.63
		DeepLabv3+ [10]with ResNet [13]	***29.04	3.06	<0.001	23	35.08
		DeepLabv3+ [10]with Xception [12]	***36.93	3.06	<0.001	30.89	42.97
		CPN [14]	***27.27	3.06	<0.001	21.23	33.31
		SOLOv2 [15]	***32.26	3.06	<0.001	26.22	38.3
		BCNet [16]	***29.1	3.06	<0.001	23.06	35.13

References

1. Wang, C.W.; Lin, K.Y.; Lin, Y.J.; Khalil, M.A.; Chu, K.L.; Chao, T.K. A Soft Label Deep Learning to Assist Breast Cancer Target Therapy and Thyroid Cancer Diagnosis. *Cancers* **2022**, *14*, 5312.
2. Lin, Y.J.; Chao, T.K.; Khalil, M.A.; Lee, Y.C.; Hong, D.Z.; Wu, J.J.; Wang, C.W. Deep Learning Fast Screening Approach on Cytological Whole Slides for Thyroid Cancer Diagnosis. *Cancers* **2021**, *13*, 3891.
3. Wang, C.W.; Liou, Y.A.; Lin, Y.J.; Chang, C.C.; Chu, P.H.; Lee, Y.C.; Wang, C.H.; Chao, T.K. Artificial intelligence-assisted fast screening cervical high grade squamous intraepithelial lesion and squamous cell carcinoma diagnosis and treatment planning. *Scientific Reports* **2021**, *11*, 1–14.
4. Khalil, M.A.; Lee, Y.C.; Lien, H.C.; Jeng, Y.M.; Wang, C.W. Fast Segmentation of Metastatic Foci in H&E Whole-Slide Images for Breast Cancer Diagnosis. *Diagnostics* **2022**, *12*, 990.
5. Wang, C.W.; Lee, Y.C.; Chang, C.C.; Lin, Y.J.; Liou, Y.A.; Hsu, P.C.; Chang, C.C.; Sai, A.K.O.; Wang, C.H.; Chao, T.K. A Weakly Supervised Deep Learning Method for Guiding Ovarian Cancer Treatment and Identifying an Effective Biomarker. *Cancers* **2022**, *14*, 1651.
6. Wang, C.W.; Chang, C.C.; Lee, Y.C.; Lin, Y.J.; Lo, S.C.; Hsu, P.C.; Liou, Y.A.; Wang, C.H.; Chao, T.K. Weakly supervised deep learning for prediction of treatment effectiveness on ovarian cancer from histopathology images. *Computerized Medical Imaging and Graphics* **2022**, *99*, 102093. https://doi.org/https://doi.org/10.1016/j.compmedimag.2022.102093.
7. Falk, T.; Mai, D.; Bensch, R.; Çiçek, Ö.; Abdulkadir, A.; Marrakchi, Y.; Böhm, A.; Deubner, J.; Jäckel, Z.; Seiwald, K.; et al. U-Net: deep learning for cell counting, detection, and morphometry. *Nature methods* **2019**, *16*, 67–70.
8. Badrinarayanan, V.; Kendall, A.; Cipolla, R. Segnet: A deep convolutional encoder-decoder architecture for image segmentation. *IEEE transactions on pattern analysis and machine intelligence* **2017**, *39*, 2481–2495.
9. Shelhamer, E.; Long, J.; Darrell, T. Fully convolutional networks for semantic segmentation. *IEEE transactions on pattern analysis and machine intelligence* **2017**, *39*, 640–651.
10. Chen, L.C.; Zhu, Y.; Papandreou, G.; Schroff, F.; Adam, H. Encoder-decoder with atrous separable convolution for semantic image segmentation. In Proceedings of the Proceedings of the European conference on computer vision (ECCV), 2018, pp. 801–818.
11. Howard, A.G.; Zhu, M.; Chen, B.; Kalenichenko, D.; Wang, W.; Weyand, T.; Andreetto, M.; Adam, H. Mobilenets: Efficient convolutional neural networks for mobile vision applications. *arXiv preprint arXiv:1704.04861* **2017**.
12. Chollet, F. Xception: Deep learning with depthwise separable convolutions. In Proceedings of the Proceedings of the IEEE conference on computer vision and pattern recognition, 2017, pp. 1251–1258.
13. He, K.; Zhang, X.; Ren, S.; Sun, J. Deep residual learning for image recognition. In Proceedings of the Proceedings of the IEEE conference on computer vision and pattern recognition, 2016, pp. 770–778.
14. Upschulte, E.; Harmeling, S.; Amunts, K.; Dickscheid, T. Contour Proposal Networks for Biomedical Instance Segmentation. *Medical Image Analysis* **2022**, p. 102371.
15. Wang, X.; Zhang, R.; Kong, T.; Li, L.; Shen, C. Solov2: Dynamic and fast instance segmentation. *Advances in Neural information processing systems* **2020**, *33*, 17721–17732.
16. Ke, L.; Tai, Y.W.; Tang, C.K. Deep Occlusion-Aware Instance Segmentation With Overlapping BiLayers. In Proceedings of the Proceedings of the IEEE/CVF Conference on Computer Vision and Pattern Recognition (CVPR), June 2021, pp. 4019–4028.
17. Szegedy, C.; Ioffe, S.; Vanhoucke, V.; Alemi, A.A. Inception-v4, inception-resnet and the impact of residual connections on learning. In Proceedings of the Thirty-first AAAI conference on artificial intelligence, 2017.
